# Immunization With Bovine Herpesvirus-4-Based Vector Delivering PPRV-H Protein Protects Sheep From PPRV Challenge

**DOI:** 10.3389/fimmu.2021.705539

**Published:** 2021-09-14

**Authors:** Daniel Rodríguez-Martín, José Manuel Rojas, Francesca Macchi, Valentina Franceschi, Luca Russo, Noemí Sevilla, Gaetano Donofrío, Verónica Martín

**Affiliations:** ^1^Centro de Investigación en Sanidad Animal (CISA-INIA), Instituto Nacional de Investigación y Tecnología Agraria y Alimentaria, Consejo Superior de Investigaciones Científicas (CSIC), Valdeolmos, Spain; ^2^Department of Medical Veterinary Sciences, University of Parma, Parma, Italy

**Keywords:** vaccine, PPRV, immune response, ruminant, protection correlates, DIVA, recombinant herpesvirus

## Abstract

The *Morbillivirus* peste des petits ruminants virus (PPRV) is the causal agent of a highly contagious disease that mostly affects sheep and goats and produces considerable losses in developing countries. Current PPRV control strategies rely on live-attenuated vaccines, which are not ideal, as they cannot differentiate infected from vaccinated animals (DIVA). Recombinant vector-based vaccines expressing viral subunits can provide an alternative to conventional vaccines, as they can be easily paired with DIVA diagnostic tools. In the present work, we used the bovine herpesvirus-4-based vector (BoHV-4-A) to deliver PPRV hemagglutinin H antigen (BoHV-4-A-PPRV-H-ΔTK). Vaccination with BoHV-4-A-PPRV-H-ΔTK protected sheep from virulent PPRV challenge and prevented virus shedding. Protection correlated with anti-PPRV IgGs, neutralizing antibodies and IFN-γ-producing cells induced by the vaccine. Detection of antibodies exclusively against H-PPRV in animal sera and not against other PPRV viral proteins such as F or N could serve as a DIVA diagnostic test when using BoHV-4-A-PPRV-H-ΔTK as vaccine. Our data indicate that BoHV-4-A-PPRV-H-ΔTK could be a promising new approach for PPRV eradication programs.

## Introduction

Peste des petits ruminants virus (PPRV) is a *Morbillivirus* that primarily affects domestic and wild ruminants. PPRV, the causal agent of peste des petits ruminants (PPR), a notifiable disease to the World Organization for Animal Health (OIE), produces considerable economic losses, predominantly in developing countries where livestock are one of the main economic resources. PPRV, like other morbilliviruses, induces immune suppression during the acute phase of the disease, which favors the establishment and aggravates the progression of secondary infections (1). Protective current PPRV vaccines are extensively used in countries where PPRV is endemic (2). They are based on attenuation of live PPRV strains (Nigeria 75/1, Sungri’96, Arasur’87, and Coimbatore’97) by serial passage in Vero cells. Single immunization with live PPRV vaccines has been able to maintain protective levels of serum antibody for up to 3 years, with antibody patterns undistinguishable from the ones generated after a natural infection. These live vaccines are, however, thermosensitive and require an efficacious cold chain to remain active, which can be problematic in PPRV-endemic countries usually situated in warm climate areas. Another drawback of live vaccines is that they cannot differentiate infected from vaccinated animals (the so-called DIVA vaccines). The DIVA approach has several advantages for vaccination programs. It allows serological surveillance of vaccinated populations. DIVA vaccines are also suited for disease control in disease-free regions that are at risk of outbreaks. It is also ideal for eradication programs in endemic regions as it permits surveillance after finishing vaccination campaigns, so that animal trade can be resumed. PPRV has been proposed as the next disease for global eradication by the OIE and the Food and Agriculture Organization of the United Nations (FAO), and thus, developing a DIVA vaccine for PPRV is highly attractive to help in this objective.

An effective viral vector-based vaccine candidate should present the expressed antigen as an immune target and should remain in the host long enough to stimulate an effective response, acting both as adjuvant and delivery system. The molecular and biological characteristics of bovine herpesvirus-4 (BoHV-4) make it an attractive vector vaccine candidate (3). It is able to replicate in a broad range of host species both *in vivo* and *in vitro* (4), yet only produces subclinical infections in cattle. The BoHV-4-A-ΔTK vector (5) is derived from BoHV-4, a non-pathogenic dsDNA virus that is able to infect different cell types *in vitro* and a variety of hosts (3, 4). The thymidine kinase (TK) gene was selected as target site for the insertion of exogenous genes for two main reasons: it is a very stable area of the virus genome and it is known not to affect viral replication *in vitro* but tend to attenuate viral replication *in vivo* (4, 6–8), thus further contributing to the safety of the vector. The recombinant BoHV-4-based viral vector is easily produced and manipulated and has a large foreign DNA accommodation capacity, being a good candidate as gene delivery vector for vaccination purposes.

Induction of potent immune responses in a range of species through recombinant BoHV-4 delivering heterologous antigens has previously been demonstrated (9). Data obtained using recombinant BoHV-4 carrying a luciferase expression cassette showed local replication at sites of immunization supporting sub-cutaneous/intramuscular route of BoHV-4-based vector inoculation for antigen production (10). The pre-existing anti-vector immunity in the target host species, which often represents one of the major concerns with vector vaccine development, is avoided with the BoHV-4 vector, as its administration does not naturally elicit the production of neutralizing antibodies. This vector has been previously successfully used as a vehicle to deliver heterologous antigens from different animal pathogens including the Nipah virus (NiV) F (fusion) and G (glycoprotein) proteins (11), the H (hemagglutinin) protein from PPRV (5), the VP2 protein from BTV (12), the Crimean-Congo nucleoprotein (N) (13), the E2 glycoprotein from bovine viral diarrhea virus (BVDV) (14), the Ebola virus GP glycoprotein (15), or the glycoprotein D from caprine herpesvirus type 1 (CpHV-1) (10). These different antigens have been shown to induce strong immunity in different animal models, such as rabbits (3) or mice (5, 12–14), but also in pigs (9, 11) or goats (15). Moreover, in challenge experiments, BoHV-4-based vectors were shown to induce a protective response against caprine herpesvirus 1 (CpHV-1) in goats (10). Even in chicken, an exogenous antigen expressed by BoHV-4-based vector induced immunity against the protein (16). Hemagglutinin (H) is a highly immunogenic PPRV envelope glycoprotein displaying both hemagglutinin and neuraminidase activities, playing a crucial role in virus attachment and penetration. Previously, a recombinant bovine herpesvirus-4 (BoHV-4)-based vector delivering an optimized PPRV-hemagglutinin expression cassette, BoHV-4-A-PPRV-H-ΔTK, was generated and assessed in immunocompetent C57BL/6 mice (5). BoHV-4-A-PPRV-H-ΔTK-immunization elicited both cellular and humoral immune responses with specific T cell, cytotoxic T lymphocyte, and serum-neutralizing antibody against PPRV detected in mice. These data indicate that recombinant BoHV-4-A-PPRV-H-ΔTK could be an effective vaccine candidate to protect against PPRV herd infection and potentially applicable for eradication programs, as it has the potential to be a DIVA vaccine.

The objectives of this study are to evaluate the immunogenicity of BoHV-4-A-PPRV-H-ΔTK vector by assessing the induction of humoral and cellular immune responses and to determine whether this vaccine protects against the disease in PPRV natural host.

## Materials and Methods

### Cells and Viruses

Ivory Coast’89 (ICV’89) PPRV strain (lineage I) and the Vero-dogSLAM cells (VDS) used to grow the virus in tissue culture were obtained from Dr. Batten and Dr. Parida (IAH, Pirbright), respectively. The virulent Morocco’08 (Mor’08) PPRV strain was kindly provided by Dr. Loutfi Chafiqua (Head of Virology Dept., Biopharma, Rabat, Morocco). VDS cells were grown in DMEM supplemented with 10% fetal bovine serum (FBS), 4 mM of L-glutamine, 1% 100× non-essential amino acids, 10 mM HEPES, 100 IU/ml of penicillin, 100 μg/ml of streptomycin, and 1 μg/ml Zeocin (all from Thermo Fisher). PPRV stocks were generated harvesting a 48–72-h VDS cell infected at a multiplicity of infection (moi) of 0.01 plaque forming units (pfu)/cell. A semipurification on a layer of 40% saccharose in TNE (10 mM Tris, 100 mM NaCl, 0.5 mM EDTA, pH 7.4) centrifuged at 120,000×*g* for 1 h was performed. Pellets were resuspended in TNE and store at −80°C. Standard plaque assay using VDS was used for PPRV titrations. PPRV was inactivated by incubation with 3 mM binary ethyleneimine (BEI) (Merck). Ovine fetal lung (OFL) cells were kindly provided by Dr. Cunha C.W. (from Washington State University, Pullman, USA), expanded in complete Eagle’s minimal essential medium (cEMEM: 2 mM of L-glutamine, 100 IU/ml of penicillin, 100 μg/ml of streptomycin, and 0.25 μg/ml of amphotericin B), supplemented with 10% FBS, and incubated at 37°C/5% CO_2_ in a humidified incubator. All the supplements for the culture medium were purchased from Gibco. BEK (bovine embryo kidney) cells (Istituto Zooprofilattico Sperimentale, Brescia, Italy; BS CL-94) and Madin–Darby bovine kidney (MDBK) cells (ATCC: CRL 6071) were maintained in cEMEM with 10% FBS and were incubated at 37°C/5% CO_2_ in a humidified incubator.

### Preparation of Recombinant Viruses in OFLs

For assessing OFL permissiveness to BoHV-4 infection, OFLs were infected at 0.1, 1, or 5 moi with BoHV-4EGFPΔTK (4) and incubated at 37°C for 2 h in cEMEM with 2% FBS. The cells were then overlaid with fresh cEMEM at 10% FBS and incubated at 37°C, 5% CO_2_. Supernatants were collected and titrated at 24, 48, and 72 h post-infection. To produce the recombinant viruses for the ovine immunization study, OFLs were infected at 0.5 moi with BoHV-4-A-PPRV-H-ΔTK (5) or BoHV-4-A-ΔTK in cEMEM with 2% of sheep serum (SS). After 3 h of incubation, the viral inoculum was removed from the cells and substituted with fresh cEMEM supplemented with 10% of SS (Biowest) and the cells were incubated at 37°C, 5% CO_2_. Forty-eight hours post-infection, the CPE was evident on the cell culture monolayer. Flasks were frozen/thawed, and the infected cells and the culture supernatant were directly harvested. The cell-associated viral particles were classically titrated on MDBK or BEK cells.

### MTT Assay

The 3-(4,5-dimethylthiazol-2-yl)-2,5-diphenyltetrazolium bromide (MTT, Sigma-Aldrich) cell metabolic assay was used to measure cell viability. Briefly, 3 × 10^3^ OFLs were seeded in 96-well plates in cEMEM and incubated for 24 h at 37°C, 5% CO_2_. Medium was refreshed with cEMEM supplemented with either 10% FBS or SS. After 24, 48, and 72 h of incubation, cells were incubated for a further 6 h with 50 μg/well of MTT before the addition of 110 μl of solubilization solution (10% SDS in HCl 0.01 M) and incubated overnight at 37°C. The optical density was measured at 620 nm in a microplate reader (Multiskan FC, Thermo Fisher Scientific). Statistical differences among treatments were tested by analysis of variance (ANOVA).

### Immunoblotting

Western immunoblotting analysis was performed on protein cell extracts from T25 cm^2^ flasks of OFL cells infected with a moi of 1 of BoHV-4-A-PPRV-H-ΔTK or mock infected. For protein extraction, 100 μl of cell extraction buffer (50 mM Tris–HCl, 150 mM NaCl, and 1% NP-40; pH 8) was added on each pellet and total protein quantification was performed using BCA Protein Assay kit (Pierce™, Thermo Fisher Scientific). Protein samples were electrophoresed on 10% SDS-PAGE and then transferred to PVDF membranes by electroblotting. The membrane was blocked in 5% skim milk (BD), incubated 1 h with primary bovine anti-gD106 monoclonal antibody (clone 1B8-F11; VRMD) diluted 1:10,000 and then probed with horseradish peroxidase-labeled anti-mouse immunoglobulin (A9044, Sigma), diluted 1:10,000, and finally visualized by enhanced chemiluminescence (Clarity Max Western ECL substrate, Bio-Rad).

### Ethical Statement

All animal experiments were carried out in a disease-secure isolation facility (BSL3) at the *Centro de Investigación en Sanidad Animal* (CISA), in strict accordance with the recommendations in the guidelines of the Code for Methods and Welfare Considerations in Behavioural Research with Animals (Directive 86/609EC; RD1201/2005), and all efforts were made to minimize suffering. Experiments were approved by the Committee on the Ethics of Animal Experiments of the Spanish *Instituto Nacional de Investigación y Tecnología Agraría y Alimentar*ia (INIA) and the *National Animal Welfare Committee* (PROEX 032/19).

### Animal Experiments

Twelve-month-old-female sheep from the “*Colmenareña*” breed from a certified provider were randomly divided into three groups, with five sheep per control group and four sheep in the vaccinated group, and housed in the same room with controlled temperature and light/dark cycles. Food and water were provided *ad libitum*. An acclimatization period of 2 weeks was observed, during which animals were monitored daily for general health status prior to the beginning of the experiment.

Animals were inoculated intramuscularly (im) with PBS (group 1; *n* = 5), with 10^6^ tissue culture infectious dose 50 (TCID_50_) of BoHV-4-A-ΔTK (group 2; *n* = 5), or with 10^6^ TCID_50_ of BoHV-4-A-PPRV-H-ΔTK (group 3; *n* = 4). One booster inoculation was performed with the same amount of recombinant virus vaccine after 21 days.

Challenge was performed at day 42 post-immunization by intravenous and intranasal inoculation of 2 × 10^7^ pfu of virulent ICV’89 PPRV strain. Animals were bled at days 0 (naive), 7, 21, 28, and 42 (pre-challenge) and at days 2, 4, 7, 9, 11, and 14 (post-challenge) and sacrificed at 14 days post-challenge (PC). Ocular, nasal, and oral swabs were collected at different days pre- and post-challenge for detection of PPRV.

After ICV’89 PPRV challenge, animals were examined daily for clinical signs of infection and their rectal temperatures recorded to ensure that any animal found to be suffering could be given appropriate veterinary care in accordance with standard veterinary practice.

Scores from 0 to 4 for each animal were calculated based on the severity of ocular, oral, and nasal congestion and discharge as well as signs of apathy, anorexia, diarrhea, and loss of appetite, using the version of the scoring method used in (17). Briefly, scores were assigned for each of the following categories: general clinical signs, pyretic response, ocular/nasal discharge, and gastrointestinal and respiratory signs. The final score obtained for each animal and day was the sum of these scores, ranging from 0 for a healthy animal to a possible maximum of 20. No animals died during the course of the experiment, and neither did they reach severity scores granting euthanasia.

Euthanasia was performed by intravenous administration of ketamine 100 mg/ml (2.2 mg/kg), atropine 1 mg/ml (0.05 mg/ml), and xylazine 20 mg/ml (0.15 mg/kg) for sedation, followed by intravenous administration of a lethal dose of pentobarbital sodium (200 mg/ml) 1 ml/1.5 kg.

A scheme of the experimental design is presented in **Table 1**.

**Table 1:** Experimental design showing different groups of animals.

Group	Vaccine	Route of vaccination	Dose of vaccine per innoculum	Virulent virus used for challenge	Route of challenge	Dose of challenge
**PBS** (n=5)	PBS	intramuscular	10^6^ TCID50	Ivory Coast'89	Intravenous and Intranasal	2x10^7^ pfu
**Control** (n=5)	BoHV-4 A-ΔTK	intramuscular	10^6^ TCID50	Ivory Coast'89	Intravenous and Intranasal	2x10^7^ pfu
**Vaccinated** (n=4)	BoHV-4 A-PPRV- H-ΔTK	intramuscular	10^6^ TCID50	Ivory Coast'89	Intravenous and Intranasal	2x10^7^ pfu

### Peripheral Blood Mononuclear Cells and Sera

Peripheral blood mononuclear cells (PBMCs) were obtained from total blood collected in EDTA directly from the jugular vein and purified by Ficoll cushion (GE Healthcare) purification as described previously (18). For serum collection, blood samples were taken into Venojet glass tubes, without anticoagulant on days 0 (naive), 7, 21, 28, and 42 (pre-challenge) and days 2, 4, 7, 9, 11, and 14 (post-challenge) and allowed to clot overnight at 4°C. On the next day, serum was obtained by centrifugation at 3,000 rpm for 10 min at 4°C, aliquoted, and stored at −80°C until use.

### Viral RNA Extraction and Quantification by qPCR

Total RNA extraction from blood samples using TRIzol (Invitrogen) and reverse transcriptions of the N-mRNAs fragments with SuperScript III reverse transcriptase (Invitrogen) were performed according to the protocols of the manufacturer. Quantifications were performed on a Mx3005 qPCR instrument (Stratagene) using the Luna I kit (NEB). Total N RNA fragment was used as standard for quantification. This was obtained as a runoff transcript from a molecular DNA clone encoding the N in the genomic sense, cloned into pGem-T Easy Vector (Promega) to provide the corresponding standard curves in the qPCR reaction. The N-ICV’89 region was amplified with primers forward-5' AGAGTTCAATATGTTATTAGCATCCAT-3' and reverse-5' TTCCCCAATCACTCTCCTCTGT-3'. Each value of the amount of PPRV RNA is the average of at least three independent determinations.

### Anti-PPRV IgG ELISA

Anti-PPRV IgG detection was performed as described previously (19). Concisely, 10^4^ pfu per well of purified PPRV Nigeria 75/1 was used to coat ELISA plates (Maxisorp, Nunc) overnight at 4°C. Blockage and washes were performed with 10% FBS in PBS and 0.1% Tween in PBS, respectively. Sera from inoculated sheep were diluted in PBS + 1% FBS prior to addition to the plate. A secondary antibody donkey anti-sheep IgG conjugated with horseradish peroxidase (Serotec) diluted 1:6,666 in PBS + 0.5% FBS was used to detect PPRV-specific IgGs. A liquid substrate system (Sigma) of 3,3',5,5'-tetramethylbenzidine (TMB) was used as a developer agent, and reactions were stopped with 3 M sulfuric acid before reading. A FLUOstar Omega (BMG Labtech) ELISA plate reader was used to read at 450 nm the optical density (OD). The measurements were made in triplicate and considered valid with standard deviations below 10% of the average. Anti-PPRV IgG titer in serum was defined as the serum dilution necessary to achieve readings twice that of pre-immune serum from the same sheep and was calculated using a linear regression of serum dilutions *vs*. OD readings at 450 nm. Data are presented as the average (±SEM) IgG titer for each treatment group.

### Virus Neutralization Test (VNT)

Serum samples were inactivated for 30 min at 56°C and tested for the presence of neutralizing antibodies as described previously (20, 21). Briefly, ICV’89 or Mor’08 PPRV stocks (100 pfu/well) were incubated with serial dilutions of inactivated sheep sera for 1 h at 37°C in 96 multiwell plates (M-96). VDS cells (2 × 10^4^ cells/well) were added to the mixtures and incubated at 37°C and 5% CO_2_. After 5 days, they were fixed with 2% formaldehyde and cells visualized by crystal violet staining. All dilutions were performed in duplicate and the neutralization titer is expressed as the reciprocal of the highest dilution of sera at which virus infection is blocked.

### PPRV Competition and Capture ELISAs

Following the instructions of the manufacturer, seroconversion in the sheep sera, based on the presence of antibodies against the nucleoprotein (N) of PPRV, was analyzed by a commercial competition ELISA [IdVet: ID Screen PPR Competition (PPRC)] (22). The commercial ELISA [IdVet: ID Screen PPR Antigen Capture (PPRAG)] was used to analyze the presence of PPRV in ocular, nasal, and oral swabs obtained in PBS from sheep at different time points.

### IFN-γ ELISPOT Assays

MSIPS4510 plates (Millipore) were used to perform ovine IFN-γ ELISPOT. Briefly, membranes were incubated overnight at 4°C with 5 μg/ml anti-bovine IFN-γ antibody (MT17.1, Mabtech, Sweden) after being activated with sterile 35% ethanol for 1 min, and washed with sterile water. The next day, they were incubated in blocking medium (RPMI supplemented with glutamine, Na+-pyruvate, HEPES, non-essential amino acids, antibiotics, and 10% FBS) for 2 h at room temperature (RT). Sheep PBMCs were then plated at a density of 2–3 × 10^5^ cells per well and incubated with BEI-inactivated ICV-PPRV, H9 peptide (from PPRV-H protein position 427–441: ITSVFGPLIPHLSGM) (10 μg/ml) (1), PBMC medium as negative control, or concanavalin-A (Con-A) (1.25 μg/ml) as positive control for 48 h at 37°C, 5% CO_2_. Cells were discarded, and after washing with PBS, membranes were incubated with biotin-labeled anti-bovine IFN-γ antibody (MT307-biotin, Mabtech, Sweden) diluted at 0.25 μg/ml in PBS + 0.5% FBS for 2 h. After five washes in PBS, membranes were incubated for 1 h with streptavidin conjugated to alkaline phosphatase (ExtrAvidin-AP, Sigma) diluted 1:10,000 in PBS + 0.5% FBS. ELISPOT assay reactions were developed using Sigma FAST BCIP/NBT (Sigma) after washing in PBS and subsequently in distilled water. IFN-γ spots were quantified with an ELISPOT plate reader (AID iSpot reader). ELISPOT assays were considered valid only when average spot counts were below 25 for control cultures and standard deviations in positive wells below 15% of the average counts. All cultures were performed in triplicates.

### PBMC Population Analysis by Flow Cytometry

The following antibodies were used to label the different PBMC subpopulations: anti-ovine CD4 (clone 44.38), CD8 (clone 38.65), and WC1 (clone 19.19), anti-human CD14 (clone TÜK4) and CD16 (clone KD1) (all from Bio-Rad), and anti-bovine B cell marker (clone BAQ44A) (Kingfisher Biotech). PBMC flow cytometry was performed as described in (1). Briefly, PBMCs were washed twice in staining buffer (PBS + 2% FBS + 0.02% sodium azide) and stained for 20 min on ice with the appropriate fluorochrome-conjugated surface antibodies. In the case of the unconjugated anti-bovine B-cell marker, a rat anti-mouse IgM FITC (clone II/41, BD Biosciences) was used as secondary antibody. Samples were run on a FACSCalibur (Becton Dickinson) flow cytometer. Dead cells were excluded from the analysis by 7-AAD staining (BD Biosciences) and gating was performed using the appropriate isotype controls. Analysis was performed using FlowJo software (TreeStar Inc.).

### Statistical Analyses

Unpaired two-tailed Student’s *t*-tests or two-way ANOVA test was used to compare treatment groups and evaluate differences. Responses to Con-A and IgG titers at different time points were compared using a Wilcoxon matched-pairs signed-rank test. Data handling analyses were performed using Prism 6.0 (GraphPad Software Inc., San Diego, CA, USA).

## Results

### BoHV-4-A-PPRV-H-ΔTK Recombinant Vaccine Was Produced in Ovine Cells to Avoid Bovine Contaminants

**Figure 1:**
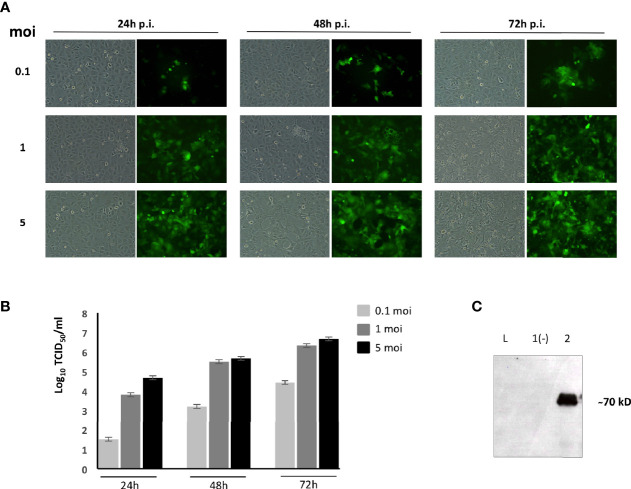
BoHV-4-A-PPRV-H-ΔTK adaptation and characterization in ovine fetal lungs (OFLs). **(A)** Representative images of BoHV-4-EFGPΔTK-infected OFLs with different moi at 24, 48, and 72 h post-infection. Magnification, ×10 (all panels). **(B)** Viral titer was measured and expressed as Log10 TCID_50_ per ml of viral particles released at 24, 48, and 72 h post-infection when infected with 0.1, 1, or 5 moi, respectively. Values shown are the means ± standard errors of three independent experiments. **(C)** Western immunoblotting of OFL cells, infected with BoHV-4-A-PPRV-H-ΔTK (lane 2) or the parental BoHV-4-AΔTK used as a negative control (lane 1). (L) Mass ladder. The lanes were loaded with the same amount of total protein.

Since the BoHV-4 vector has a bovine origin and we wanted to immunize sheep, we adapted our current protocol of recombinant vector production to suit the immunization target species. First, we established a protocol to produce BoHV-4 vectors free of bovine antigens. BoHV-4 vectors are typically propagated in bovine cells and in the presence of FBS. Thus, a protocol to grow BoHV-4 recombinant viruses in a permissive ovine cell line in the absence of FBS was established. For this purpose, the recombinant BoHV-4 vector has been produced following a specific infection protocol on the permissive OFL cell line in the absence of FBS. OFLs proved to be totally permissive to recombinant BoHV-4-EGFP-ΔTK infection (**Figure 1A**) reaching a good titer (between 10^6^ and 10^7^ TCID_50_/ml) (**Figure 1B**) and to efficiently express PPRV-H antigen when infected with the BoHV-4-A-PPRV-H-ΔTK recombinant vector, as shown by Western blot analysis (**Figure 1C**). Furthermore, MTT analysis of OFLs cultured in sheep serum showed no detrimental effect on cell growth compared with that in the presence of FBS (**Supplementary Figure S1**). Therefore, in this protocol, OFLs were expanded in a medium with 10% of FBS until they reached 90% of confluence, then infected with BoHV-4-A-PPRV-H-ΔTK and BoHV-4-A-ΔTK at low moi (0.5) in media containing 2% sheep serum. Three hours post-infection, viral inoculum was substituted with cEMEM containing 10% sheep serum. CPE appeared on OFL monolayers at 48 h post-infection. Cell-associated virus particles were then collected and titrated (**Supplementary Figure S2**). This protocol allowed the production of high titers (5 × 10^6^ TCID_50_/ml) of BoHV-4-A-PPRV-H-ΔTK and BoHV-4-A-ΔTK free of xenogenic bovine antigens.

### BoHV-4-A-PPRV-H-ΔTK Vaccination Protects Against PPRV Challenge

In order to study the protective efficacy of the potential BoHV-4-A-PPRV-H-ΔTK vaccine, the following experiment was set up. Two different control groups of sheep were inoculated intramuscularly with PBS (*n* = 5) or the empty vector BoHV-4-A-ΔTK (*n* = 5), and four animals received the potential vaccine, BoHV-4-A-PPRV-H-ΔTK (**Table 1**). All groups received a boost 21 days later. The challenge was performed at day 42 post-immunization with the virulent ICV’89 PPRV strain.

**Figure 2:**
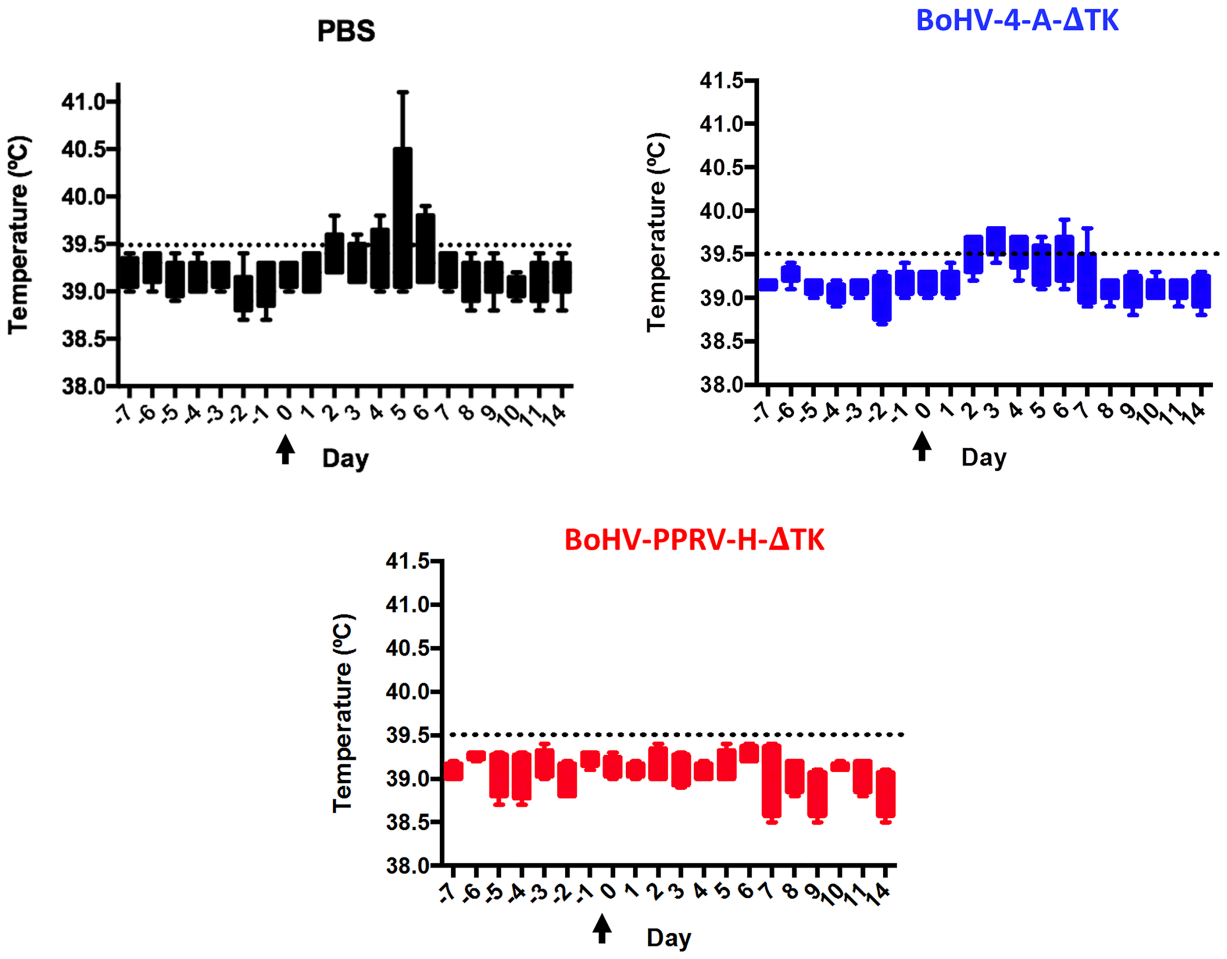
Rectal temperature in immunized sheep. The temperatures from day 38 (day −7) of the experiment to day 14 PC are represented in a box and whiskers plot that shows the minimum to maximum values in a box. As indicated, each panel corresponds to each experimental group of animals. Arrows indicate the day of ICV’89 PPRV challenge (day 0). The threshold temperature value above which animals were considered to have fever (39.5°C) is indicated as a dashed line in each panel.

**Figure 3:**
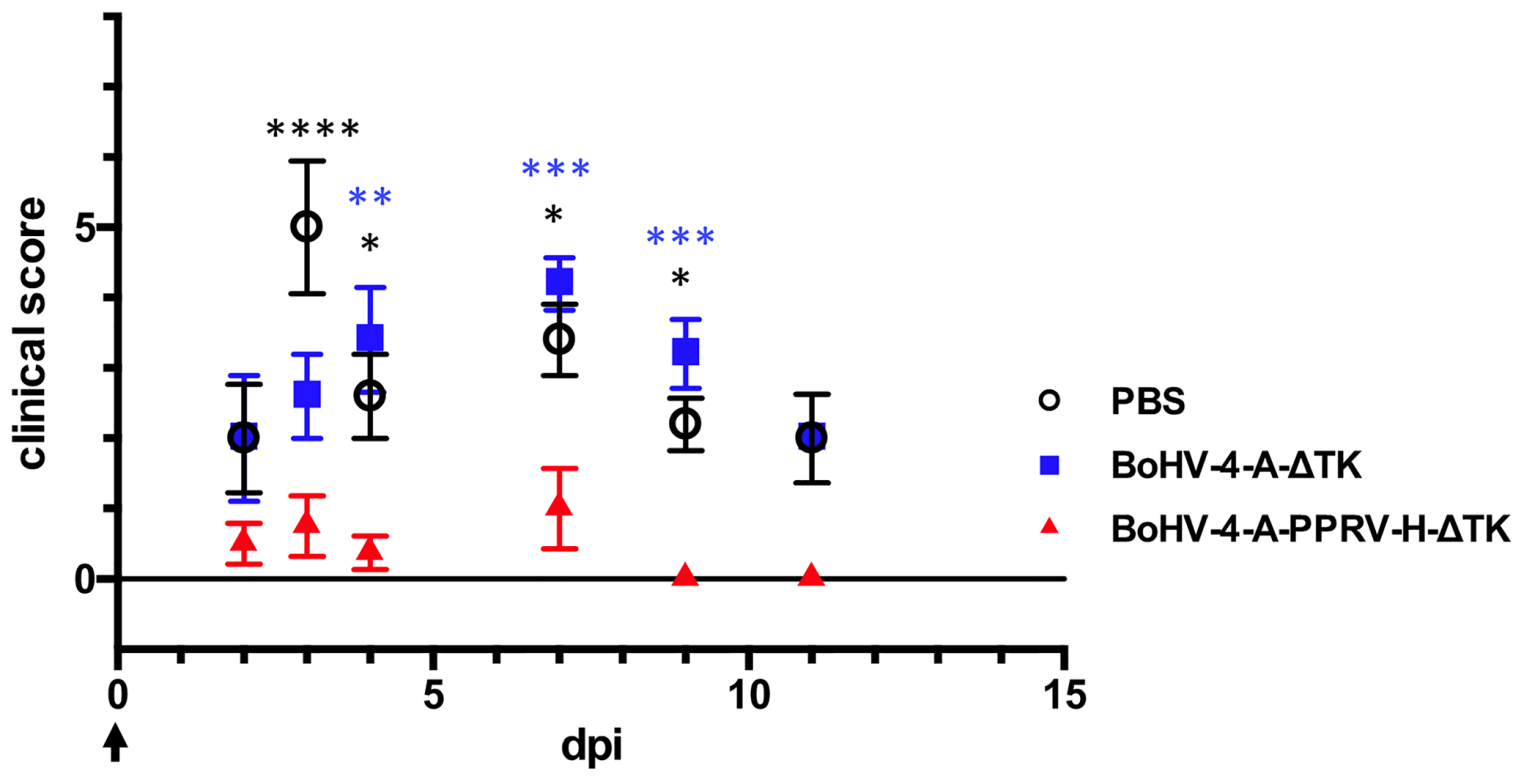
Clinical disease of immunized sheep following PPRV challenge. Following the clinical score proposed on (23) with slight modifications (17), the graded clinical observation for the development of disease after PPRV challenge in immunized sheep was represented. Data are presented as average (± SEM) score for different days post-challenge (dpi) for each treatment group as indicated in the legend. Differences were found to be significant (*p < 0.05; **p < 0.005; ***p < 0.0005; ****p < 0.00005; two-way ANOVA) between control (PBS or BoHV-4-A-ΔTK) and vaccinated group from days 3 to 8 post-challenge.

Daily rectal temperature and clinical signs were monitored in all experimental sheep groups during the trial to determine the effectivity of the BoHV-4-A-PPRV-H-ΔTK potential vaccine. Normal temperature values ranged between 38 and 39.5°C, as observed in all animals before challenge, and were not affected by BoHV-4-A-ΔTK vector inoculations (**Figure 2**). Sheep in the two control groups (PBS and BoHV-4-A-ΔTK groups) developed pyrexia (rectal temperature above 39.5°C) between days 2 and 7 after challenge (**Figure 2**, control groups, black and blue), which contrasted strongly with all vaccinated sheep (BoHV-4-A-PPRV-H-ΔTK group) that maintained normal temperature values (**Figure 2**, red). Clinical signs of disease including high temperature, conjunctivitis, moderate mucopurulent nasal discharge, poor appetite, diarrhea, dull look of wool, and mild depression were detected in unvaccinated animals (PBS or BoHV-4-A-ΔTK). By contrast, clinical signs were strongly reduced or absent in vaccinated sheep (**Figure 3**). These data indicate that sheep vaccinated with BoHV-4-A-PPRV-H-ΔTK were protected against virulent PPRV challenge.

### BoHV-4-A-PPRV-H-ΔTK Vaccination Prevents PPRV Shedding From Infected Sheep

PPRV transmission is due mainly to the secretions of the infected animals in contact with healthy ones. Thus, the ocular, nasal, and oral swabs were tested for the presence of PPRV using a commercial ELISA capture procedure (c-ELISA) (**Table 2**). Every single control animal showed PPRV-positive swabs between days 2 and 11 PC. By contrast, all the swabs collected from vaccinated sheep remained negative for PPRV at all assayed time points, even while these animals were in direct contact with PPRV-infected sheep from the control group. Thus, the BoHV-4-A-PPRV-H-ΔTK vaccine candidate impaired the secretion of virus from vaccinated animals after challenge and prevent contagion from infected animals.

**Table 2:** PPRV c-ELISA detection.

Group	D42^a^	D2PC^b^	D4PC^b^	D7PC^b^	D11PC^b^
Swab	C^c^N^d^O^e^	C^c^N^d^O^e^	C ^c^N^d^O^e^	C ^c^N^d^O^e^	C ^c^N^d^O^e^
**PBS**	**- - -**	**- - -**	**- + -**	**- + -**	**- - -**
	**- - -**	**- - -**	**- + -**	**- + -**	**- - -**
	**- - -**	**- + -**	**+ - -**	**- + +**	**+ - -**
	**- - -**	**- - +**	**-- -**	**+ + +**	**- + -**
	**- - -**	**- - -**	**+- -**	**+ + +**	**- + +**
**Control**	**- - -**	**- - -**	**- - -**	**- + -**	**- - -**
	**- - -**	**- - -**	**- - -**	**- + -**	**- - -**
	**- - -**	**- - -**	**- + +**	**- + +**	**- - +**
	**- - -**	**- - -**	**- - -**	**+ + +**	**- + +**
	**- - -**	**- - -**	**- - -**	**+ + +**	**+ + -**
**Vaccinated**	**- - -**	**- - -**	**- - -**	**- - -**	**- - -**
	**- - -**	**- - -**	**- - -**	**- - -**	**- - -**
	**- - -**	**- - -**	**- - -**	**- - -**	**- - -**
	**- - -**	**- - -**	**- - -**	**- - -**	**- - -**

^+^PRV-positive samples. ^−^PPRV-negative samples.

a Sera obtained from non-infected sheep at 21 days post-boost (42 days after first immunization).

b Sera obtained from PPRV-challenged animals at the indicated days post-challenge.

c C, conjunctiva swab.

d N, nasal swab.

e O, oral swab.

### PPRV Replication Is Abrogated in BoHV-4-A-PPRV-H-ΔTK-Vaccinated Sheep

**Figure 4:**
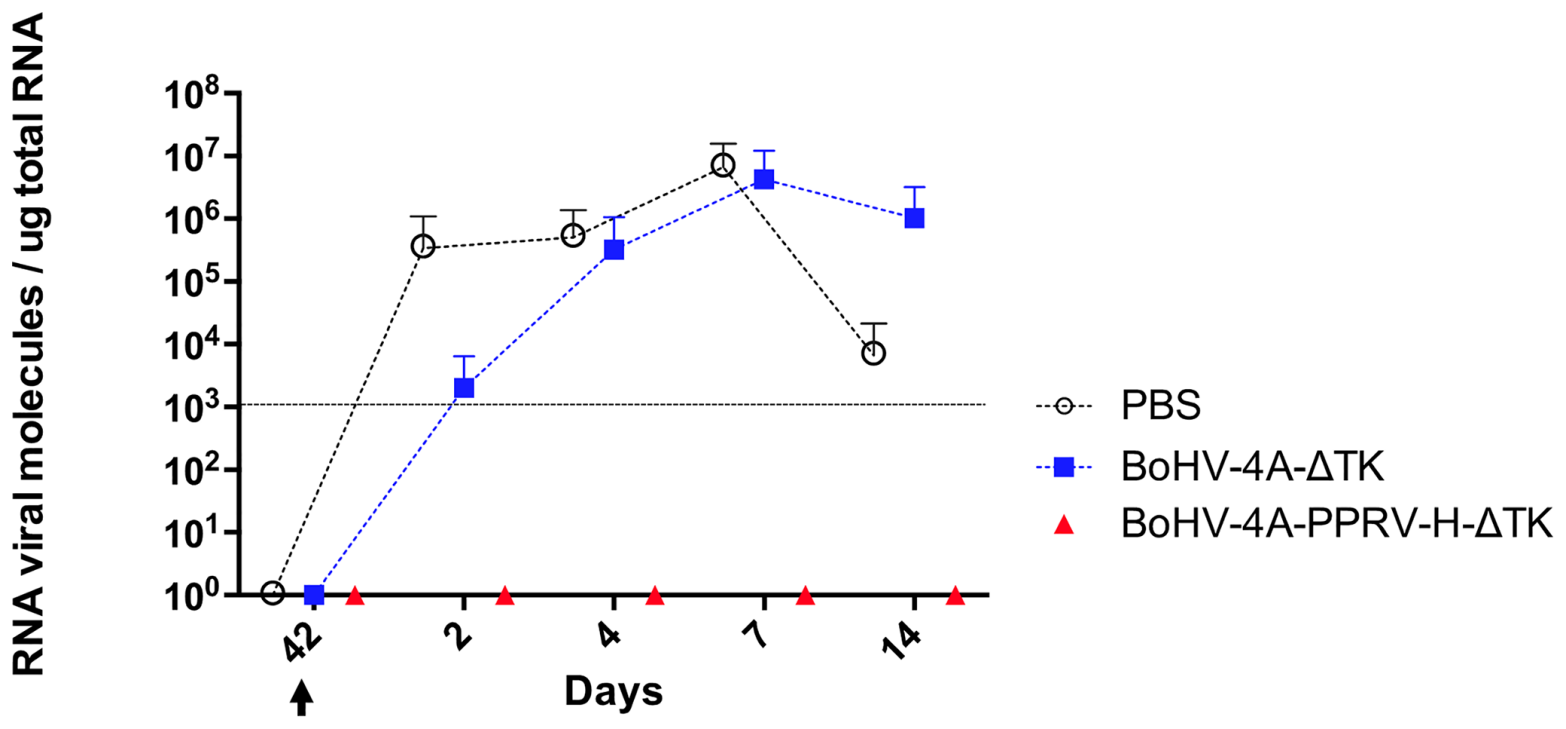
Quantification of viral RNA molecules in vaccinated sheep. PPRV RNA molecules were quantitated by qPCR as described in the *Materials and Methods*. The data are represented as the mean of the RNA values obtained for the animals of the same treatment group per microgram of total blood RNA for the indicated days. The different treatment groups are indicated in the legend [PBS (black), control vector (BoHV-4-A-ΔTK) (blue), or BoHV-4-A-PPRV-H-ΔTK (red)]. Triplicates for each sample were used. Differences were found to be significant (*p* < 0.05; Mann–Whitney test) between control (PBS or BoHV-4-A-ΔTK) and vaccinated groups.

A quantitative real-time of the PPRV-N gene was used to determine whether PPRV replication is compromised in vaccinated sheep (**Figure 4**). The number of N-PPRV messenger RNA was determined from blood samples obtained at different times points. As seen in the graph, in both control groups (PBS and BoHV-4-A-ΔTK), viral RNA molecules were detected as early as day 2 PC and increased over the following days up to 7 PC and still being detectable at day 14 PC. However, in the group of vaccinated sheep, no viral RNA was detected at any of the time points analyzed. This indicates that sheep vaccination with recombinant BoHV-4-A-PPRV-H-ΔTK totally impairs replication of PPRV after challenge.

### Vaccination With Recombinant BoHV-4-A-PPRV-H-ΔTK Virus Induces PPRV-Specific IgG and Neutralizing Antibodies in Sheep

**Figure 5:**
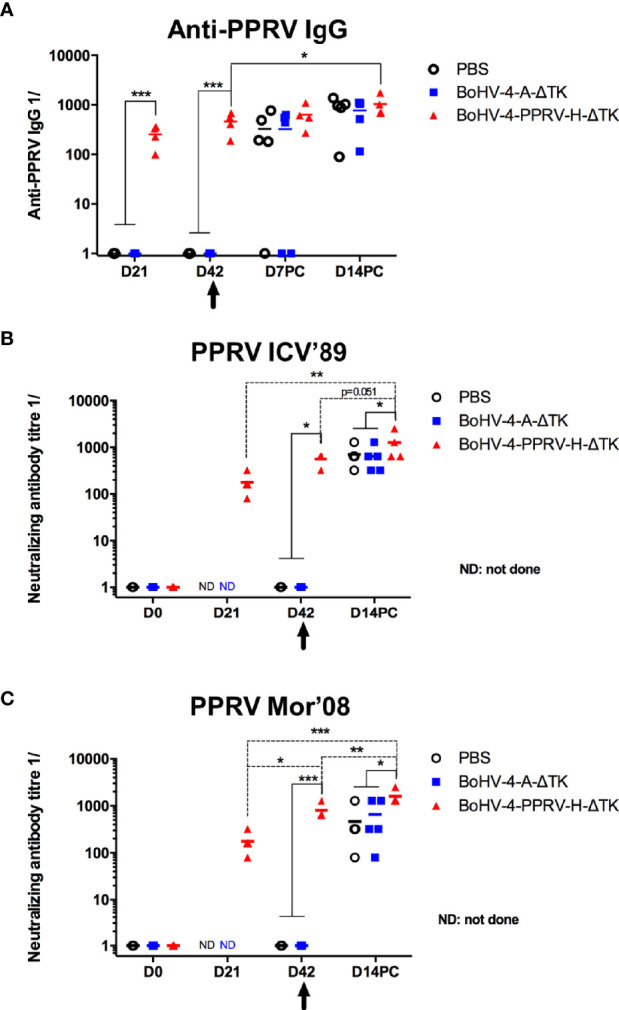
Vaccination with recombinant BoHV-4-A-PPRV-H-ΔTK virus expressing the H protein induces PPRV-specific and neutralizing IgG in sheep. **(A)** Groups (*n* = 5/5/4) of sheep were inoculated intramuscularly with PBS (black), control vector (BoHV-4-A-ΔTK) (blue), or BoHV-4-A-PPRV-H-ΔTK (red) at days 0 and 21, and at day 42 (indicated by the arrow), all animals were challenged with virulent PPRV ICV’89. At the indicated time points, the serum samples obtained were analyzed for PPRV-specific IgG by ELISA using ICV’89 PPRV-coated plates. Data are presented as 1/X IgG titer for each animal. *p < 0.05 day 14 PC (post-challenge) *vs*. day 42 in immunized sheep (paired *t*-test). ***p < 0.001 in vaccinated (BoHV-4-A-PPRV-H-ΔTK) *vs*. control (PBS and BoHV-4-A-ΔTK) sheep at the same time point (one-way ANOVA with Fisher LSD post-test). **(B, C)** Neutralizing antibodies titers against **(B)** PPRV ICV’89 or **(C)** PPRV Mor’08 were determined for the same animal groups described in **(A)** and expressed as the reciprocal of the last dilution of serum that blocked 50% of the virus-specific cytopathic effect in flat bottom 96 plates. **(B)** *p < 0.05 in vaccinated (BoHV-4-A-PPRV-H-ΔTK) *vs*. control (PBS and BoHV-4-A-ΔTK) sheep at the same time points, at day 42 (before challenge) and at day 14 PC (one-way ANOVA with Fisher LSD post-test). **p < 0.01 day 14 PC *vs*. day 21 in immunized sheep and *p* = 0.051 at day 14 PC *vs*. day 42 in immunized sheep (paired *t*-test). **(C)** *p < 0.05; ***p < 0.001 in vaccinated (BoHV-4-A-PPRV-H-ΔTK) *vs*. control (PBS and BoHV-4-A-ΔTK) sheep at the same time points, i.e., at day 42 (before challenge) and at day 14 PC (one-way ANOVA with Fisher LSD post-test). *p < 0.05 day 21 *vs*. day 42; ***p < 0.001 day 21 *vs*. day 14PC and **p < 0.01 at day 42 *vs*. day 14PC in immunized sheep (paired *t*-test).

To study the correlates of immunity induced by the vaccine with the observed protection, we next analyzed the generation of PPRV-specific IgGs by ELISA (**Figure 5A**). PPRV-specific IgGs developed in all vaccinated sheep at day 21 post-priming, with almost duplicated titer values after the booster at day 42 post-immunization and with a further slight increase at days 7 and 14 after the viral challenge. Conversely, none of the control sheep sera contained IgGs against PPRV before challenge. Sera obtained at different times PC showed PPRV-specific IgG titers in control animals as expected, which remained below the titers obtained in the vaccinated group. PPRV protection was related with the level of the neutralizing antibodies present in serum. Sera were analyzed in a virus neutralization test using the ICV’89 PPRV strain used for challenge. As reflected in **Figure 5B**, the antibodies from vaccinated animals neutralized the lineage I ICV’89 PPRV infection before (days 21 and 42) and after challenge (day 14 PC) with increasing neutralization titers. We also assessed the cross-reactivity of the neutralizing antibodies induced by BoHV-4-A-PPRV-H-ΔTK immunization using the virulent PPRV Mor’08 strain, which belongs to lineage IV (**Figure 5C**). Vaccination was capable of inducing neutralizing antibodies that blocked PPRV Mor’08 infections, which shows that BoHV-4-A-PPRV-H-ΔTK immunization elicits neutralizing antibodies that cross-react with different PPRV lineages. Neutralizing activity in control animals was detected at day 14 PC, with statistically lower values as compared with those detected in the vaccinated animals, which indicated that vaccination increased neutralizing antibody responses.

### BoHV-4-A-PPRV-H-ΔTK Vaccination in Sheep Is a DIVA Vaccine

To determine whether BoHV-4-A-PPRV-H-ΔTK is a DIVA vaccine, sera from vaccinated sheep were tested for the presence of antibodies against the PPRV-N protein, using a commercial competitive ELISA assay. Sera from the vaccinated sheep group did not present anti-N antibodies before challenge (**Table 3**). After PPRV challenge, all control animals presented antibodies against N from days 4 to 14. In contrast, vaccinated animals showed a delay in seroconversion, with anti-N antibodies becoming detectable only at day 14 post-challenge. Thus, the presence of antibodies against PPRV-H protein (**Figure 5**) in the absence of anti-N antibodies could serve as a DIVA diagnostic test when using BoHV-4-A-PPRV-H-ΔTK as vaccine.

**Table 3:** PPRV N antigen seroconversion.

Group	D0^a^	D42^b^	D2PC^c^	D4PC^c^	D7PC^c^	D14PC^c^
**PBS**	–	–	–	+	+	+
	–	–	–	+	+	+
	–	–	–	+	+	+
	–	–	–	+	+	+
	–	–	–	+	+	+
**Control**	–	–	–	+	+	+
	–	–	–	+	+	+
	–	–	–	+	+	+
	–	–	–	+/-	+	+
	–		–	+	+	+
**Vaccinated**	–	–		–	+/-	+
	–	–	–	–	+/-	+
	–	–	–	–	+/-	+
	–	–	–	–	+/-	+

Sera obtained from ^a^non-vaccinated sheep at day 0, ^b^sheep at 21 days post boost (42 days post first immunization) or from ^c^PPRV challenged animals at the indicated days. Following manufacturers protocols:

+: Anti-N Positive samples.

+/-: Anti-N Doubtful samples.

-: Anti-N Negative samples.

### Specific PPRV T-Cell Responses Are Triggered by BoHV-4-A-PPRV-H-ΔTK in Vaccinated Sheep

**Figure 6:**
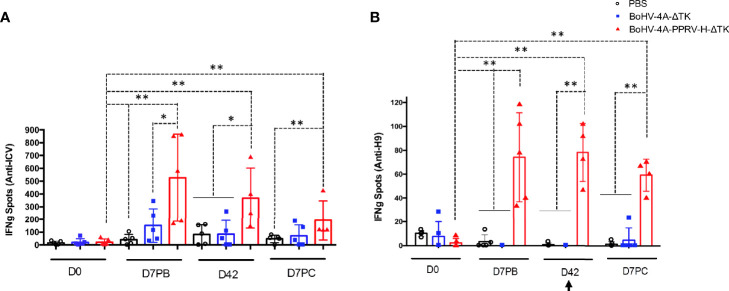
Specific IFN-γ production to ICV’89 PPRV strain and H9 peptide in PBMCs from BoHV-4-A-PPRV-H-ΔTK-inoculated sheep detected by ELISPOT assay. PBMCs from sheep inoculated with PBS (black), control vector (BoHV-4-A-ΔTK) (blue), or BoHV-4-A-PPRV-H-ΔTK (red) were isolated at days 0 and 7 post-boost (PB), day 42 post-immunization, and day 7 post-challenge (PC) and cultured for 48 h in the presence of BEI-inactivated ICV’89 PPRV strain virus **(A)** or H9 peptide from PPRV-H protein **(B)**. The production of IFN-γ was measured using an ELISPOT assay. Data are presented as average (± SEM) IFN-γ spots above background for 10^6^ cells in each treatment group. A positive control of PBMCs activated with 1.25 μg/ml Con-A (Sigma) was always included to validate the ELISPOT assay. *p < 0.05 and **p < 0.005 Mann–Whitney test. The black arrow (D42) denotes the time of virulent PPRV ICV’89 challenge in all animals.

BoHV-4-A-PPRV-H-ΔTK is capable of generating a humoral response endorsed by high values of neutralizing antibodies detected before and after the challenge, limits viral replication, and prevents virus shedding. Subsequently, the activation of cellular immune responses was evaluated through the specific anti-PPRV IFN-γ production by PBMCs from vaccinated sheep (**Figure 6**). PBMCs from all animals at days 0 and 7 post-boost (PB), 21 post-boost (D42), and day 7 PC were stimulated with inactivated PPRV or the H9 peptide (1) from the PPRV-H protein. Cells obtained from animals of the BoHV-4-A-PPRV-H-ΔTK-vaccinated group produced IFN-γ against PPRV at day 7 PB, which was maintained throughout the time points assessed in this experiment (D42 and D7 PC) (**Figure 6A**). Moreover, cellular response to the H9 peptide in vaccinated sheep was also detected at the same time points (**Figure 6B**). None of the control animals (PBS or BoHV-4-A-ΔTK) showed IFN-γ production against PPRV or H9 peptide at any of the time points assessed (**Figures 6A, B**), even after the PPRV challenge. These data demonstrate that specific PPRV T-cell responses were triggered in BoHV-4-A-PPRV-H-ΔTK-vaccinated sheep.

**Figure 7:**
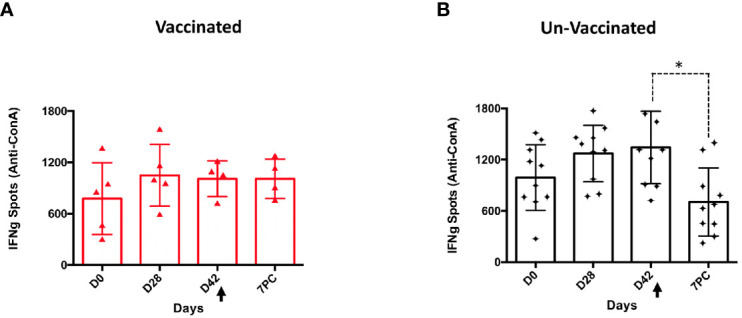
Immunosuppression in PPRV-infected sheep. T cells responding to Con-A stimulation producing IFN-γ at days 0, 28, and 42 post-vaccination and day 7 after challenge in BoHV-4-A-PPRV-H-ΔTK-vaccinated sheep **(A)** and control sheep (PBS and BoHV-4-A-ΔTK) **(B)**. Each dot corresponds to the average IFN-γ spot count for 10^6^ cells in one animal. Bars indicate the average of all animals. A Wilcoxon matched-pairs signed-rank test was used for statistical analysis (*p < 0.05). The black arrow (D42) denotes the time of virulent PPRV ICV’89 challenge in all animals.

The lack of T-cell responses in unvaccinated sheep, even after challenge (17), could reflect the immunosuppression attributed to the acute phase of PPRV disease (24). To determine T-cell functionality in PPRV-challenged sheep, T-cell stimulation with the mitogen Con-A was evaluated. In control sheep, which received either PBS or BoHV-4-A-ΔTK, the production of IFN-γ in response to Con-A stimulation was significantly reduced at day 7 post-challenge compared with before challenge (D0, D28, and D42) (*p* < 0.05; Wilcoxon matched-pairs signed test) (**Figure 7B**). By contrast, sheep vaccinated with BoHV-4-A-PPRV-H-ΔTK responded similarly to Con-A before and after the viral challenge indicating that vaccination was capable of overcoming the immunosuppressive effects of the virus (**Figure 7A**).

### BoHV-4-A-PPRV-H-ΔTK Vaccination Results in CD4^+^ and CD8^+^ T-Cell Expansion Post-PPRV Challenge

**Figure 8:**
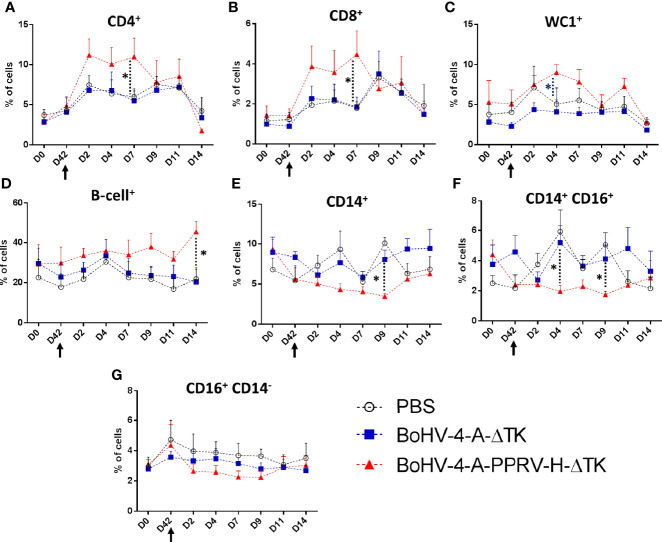
Effect of BoHV-4-A-PPRV-H-ΔTK vaccination in ovine PBMC populations. PBMCs obtained from the three treatment groups [PBS (black), BoHV-4-A-ΔTK (blue), and BoHV-4-A-PPRV-H-ΔTK (red)] were stained with different monoclonal antibodies and analyzed by flow cytometry at different time points [pre-immunization (D0), pre-challenge (D42), and days 2, 4, 7, 9, 11, and 14 post-challenge (PC)]. Average percentage of **(A)** CD4^+^ cell population, **(B)** CD8^+^ cell population, **(C)** WC1^+^ (γδ T cell) population, **(D)** B-cell^+^ population, **(E)** CD14^+^ cell population, **(F)** CD14^+^ CD16^+^ cell population, and **(G)** CD16^+^ CD14^−^ cell population for each sheep group were plotted. *p < 0.05 one-way ANOVA test (BoHV-4-A-PPRV-H-ΔTK *vs*. BoHV-4-A-ΔTK and PBS control groups). The black arrow (D42) denotes the time of virulent PPRV ICV’89 challenge in all animals.

To further investigate the immune correlates with the protection offered by BoHV-4-A-PPRV-H-ΔTK vaccination, an analysis of the main cell populations present in PBMC was performed in all three groups of treated sheep at different time points by flow cytometry. Thus, PBMCs were isolated and stained with anti-CD4, CD8, B-cell, WC1 (γδ T cells), CD14 and CD16 (NK cells), CD14 (monocytes), and CD14 and CD16 (non-classical monocytes)-specific antibodies, and the percentage of cell populations were determined (**Figure 8**). No significant changes in cell populations were observed after vaccination day 0 (pre-immunization) *vs*. day 42 (pre-challenge). A steeper increase in CD4^+^ and CD8^+^ T cells in the BoHV-4-A-PPRV-H-ΔTK-vaccinated group was observed as soon as day 2 PC and maintained until day 7 PC when compared with the control groups vaccinated with PBS or BoHV-4-A-ΔTK (**Figures 8A, B**). Both control groups showed a clear CD8^+^ T-cell expansion at day 9 PC (**Figure 8B**) which could be consistent with the onset of a primary immune response to the infection. A similar WC1^+^ (γδ T cells) response is observed in vaccinated sheep with a significant expansion with respect to the control groups at day 4 (**Figure 8C**). A statistically significant increase in B cells in BoHV-4-A-PPRV-H-ΔTK-vaccinated sheep (**Figure 8D**) was also detected at day 14 PC when compared with PBS- or BoHV-4-A-ΔTK-vaccinated animals. PPRV infection also appeared to increase monocyte populations (both in total CD14^+^ monocytes and in CD14^+^ CD16^+^ monocytes) at days 4 and 9 PC in PBS- and BoHV-4-A-ΔTK-inoculated animals when compared with BoHV-4-A-PPRV-H-ΔTK-immunized sheep (**Figures 8E, F**). No statistically significant differences are found between vaccination groups for NK cells (CD14^−^ CD16^+^) (**Figure 8G**). Overall, these data indicated that BoHV-4-A-PPRV-H-ΔTK immunization led to a rapid expansion of T cells following PPRV challenge.

## Discussion

In the present work, we describe that vaccination with BoHV-4-A-PPRV-H-ΔTK protects sheep against virulent PPRV ICV’89 challenge. Vaccinated animals presented no pyrexia, little to no clinical signs of infection, and no viremia as measured by RT-qPCR. More importantly, we could not detect PPRV presence in ocular, nasal, and oral swabs in vaccinated animals indicating that vaccination impaired virus shedding. This shows that BoHV-4-A-PPRV-H-ΔTK vaccination has the potential to limit the spread of this highly transmissible disease. This is further confirmed by the late seroconversion (tested with an anti-N-based ELISA assay) in vaccinated sheep when compared with control animals, which suggests that virus replication is reduced in these animals. Besides, vaccinated animals are protected even while they have been in close contact with unvaccinated sheep, which became ill due to PPRV infection. Moreover, the detection of anti-PPRV IgG prior to challenge in vaccinated animals indicates that a DIVA test based on differentiating immunity to PPRV-H and PPRV-N protein could be developed. Vaccinated animals would only generate antibodies against H, whereas infected animals would generate antibodies against both H and N. BoHV-4-A-PPRV-H-ΔTK has therefore the potential to become a DIVA vaccine for PPRV.

Although an ovine herpesvirus-2 glycoprotein (25) and a bovine viral diarrhea virus glycoprotein E2 (14) have already been expressed using the BoHV-4-A-ΔTK vector and delivered in sheep, we successfully describe in this work, for the first time, the efficacy of BoHV-4-based vector in terms of vaccination in sheep. Because PPRV mainly replicates in the respiratory tract, an interesting approach to analyze in future vaccination trials against PPRV would be the intranasal antigen delivery, which has already been used successfully with the BoHV-4 vector in cattle (26).

In sheep, replication of BoHV-4-derived vaccine vectors is restricted to the inoculum site, where antigen expression occurs (10). Moreover, since BoHV-4 does not naturally elicit neutralizing antibody production in its target host, the potential drawback of pre-existing anti-vector immunity is avoided. Our data show that immunization with recombinant BoHV-4 vectors is safe in sheep, an animal with phylogenetic proximity to the target species of the vector, as no adverse effects were observed after inoculation with either BoHV-4-A-ΔTK or BoHV-4-A-PPRV-H-ΔTK. To enhance sheep immune response, vaccine inocula were produced in an ovine culture system. Virally vectored vaccines are commonly produced on cell monolayers and in the presence of serum from animal species different from the immunization target and often require a purification step for vaccine preparation. The purification step is usually essential to reduce competition between the delivered antigen and the typically abundant amount of xenoantigens from cells and serum. The BoHV-4-based vaccine described herein has been produced following a specific culture protocol aimed at eliminating any bovine antigens. Thus, the BoHV-4 vector was successfully produced at high titer (10^6^–10^7^ TCID_50_/ml) in an ovine cell line in the presence of sheep serum rather than FBS. The simplified protocol developed for viral vector propagation can be used for a rapid scale-up and low-cost vaccine preparation, which is highly necessary in the case of immunization programs for potential epizootic circumstances.

One of the advantages of herpes viral vectors is that they stimulate both humoral and cellular responses, as shown in this work where the immunization with BoHV-4-A-PPRV-H-ΔTK triggers the production of anti-PPRV IgGs, neutralizing antibodies and IFN-γ-secreting cells. These components of adaptive immunity probably mediate protection against virulent PPRV challenge in vaccinated sheep. The correlates of immunity with protection against PPRV are not fully defined. Typically, during viral infection, both effector arms of adaptive immunity contribute to protection: cellular immunity mediated by cytotoxic T lymphocytes helps clear infected cells, while neutralizing antibodies prevent re-infection. Helper T-cell activation also plays an intrinsic part in finely tuning the antibody repertoire to the pathogen. In PPRV, antibody-dependent cell-mediated cytotoxicity (ADCC) is also likely to contribute to disease clearance (27). Both arms of adaptive immunity thus represent complementary weaponry that should be elicited by PPRV vaccine candidates. Overall, our data demonstrate the potency of the recombinant BoHV-4-A-PPRV-H-ΔTK construct as a PPRV vaccine. Even before the booster, neutralizing antibody titers against PPRV induced by BoHV-4-A-PPRV-H-ΔTK were well above the minimum levels titer of 10 estimated by the OIE to be protective against PPRV infection. Moreover, the rapid expansion of CD8^+^ and CD4^+^ T cells after PPRV challenge (detected from day 2 post-challenge) suggests that BoHV-4-A-PPRV-H-ΔTK immunization led to differentiation of memory anti-PPRV T cells. Similar to vaccination with recombinant adenovirus expressing PPRV-H or PPRV-F proteins (1), BoHV-4-A-PPRV-H-ΔTK can trigger immunity to T-cell epitopes presented during the course of PPRV infection. We also detected an expansion of γδ T cells in vaccinated animals at day 4 post-challenge. γδ T cells represent an important portion of circulating T cells in ruminants, and they can be implicated in antiviral immunity [reviewed in (28)]. Their role in anti-PPRV responses is, however, unknown. Their increase after challenge in vaccinated sheep indicates that they could participate in anti-PPRV immunity. Further work will be required to elucidate the role of these cells in this viral infection. A more in-depth analysis of these anti-PPRV responding T cells would be useful in the future to elucidate their precise phenotype. Further analysis of the duration of immunity induced by this vaccination will also be helpful.

Ideally, a PPRV vaccine candidate should also elicit immunity to multiple PPRV lineages. Current live-attenuated vaccines usually achieve this cross-reactivity, and as such, a new vaccine candidate should aim at providing similar benefits. The BoHV-4-A-PPRV-H-ΔTK vector was constructed with an H gene derived from the vaccine strain Nig’75 (lineage II). We have found that immunization with this vector led to the production of neutralizing antibodies that cross-reacted with PPRV lineages I and IV and protected against ICV’89 strain (lineage I) challenge in sheep. This indicates that BoHV-4-A-PPRV-H-ΔTK could provide protection against a broad range of PPRV strains. Cellular immunity is often another component of the adaptive immunity that confers cross-protection against multiple viral strains. For instance, in BTV infections where cross-protection among serotypes is limited, vaccination strategies that elicit cellular immunity to the more conserved proteins of the virus resulted in protection against heterologous serotypes (27, 29). Thus, vaccine vectors that effectively deliver T-cell antigens are very attractive constructs to promote multiserotype immunity. BoHV-4 and adenovirus vectors are particularly suited for T-cell antigen delivery as they can express high intracellular amounts of the transgene products and thus could, for instance, be used with minigene cassettes that express T-cell epitopes from multiple viral antigens. BoHV-4-A-PPRV-H-ΔTK immunization also elicited cellular immunity against PPRV, which further suggests that it could provide protection against a wide spectrum of PPRV strains.

An important aspect related to PPRV pathogenesis resides in its immunosuppressive effects. Indeed, PPRV is devastating not only because of the direct effects of the infection, but also because it weakens immunity that allows for opportunistic pathogens to worsen the outcome of the infection. A drawback of the currently employed live-attenuated PPRV vaccines is that they retain immunosuppressive potential, which is not ideal (24). In our experimental setting, we detected impaired cellular responses to the T-cell mitogen Con-A in infected control sheep illustrating the immunosuppressive effects of the infection. As observed during vaccination using a human recombinant adenovirus 5 expressing the PPRV-H (17), vaccination with the BoHV-4-A-PPRV-H-ΔTK abrogated the immunosuppressive effect of PPRV infection per se. Thus, usage of recombinant viruses may eliminate the state of susceptibility to secondary infections induced by conventional vaccination against PPRV.

We also found that PPRV infection affected monocyte populations, an effect that was not observed in vaccinated animals. We detected an increase in CD14^+^ monocytes in control groups between days 4 and 9 after challenge. More precisely, our data show that the circulating CD14^+^ CD16^+^ monocyte fraction increases at these time points probably because of the infection. In human, CD14^+^ CD16^+^ monocytes are classified as intermediate/non-classical monocytes that can typically stimulate T-cell proliferation and produce proinflammatory cytokines [reviewed in (30)]. Similar functions for these cells have been described in cattle [reviewed in (31)] and their presence has been confirmed in sheep (32) although little is known about the functions of this monocyte population in this species. Our data indicate that CD14^+^ CD16^+^ monocytes could be involved in the primary response to PPRV infection, since the frequency of this monocyte subset was unchanged in vaccinated sheep but increased in control sheep. Further work will be required to further characterize this monocyte population in sheep and determine whether they share similar function to their human or cattle counterparts during immune responses.

The determination of the precise antigen formulation necessary for PPRV protection remains an open question in the field. From the present study and others, it appears that PPRV-H could be an important determinant for the induction of protective immunity against PPRV (33). Delivering this PPRV antigen to the host immune system through recombinant viral vectors, in PPRV virus-like particles (VLPs), or using an anti-H idiotypic DNA vaccine has proved effective in triggering cellular and humoral immunity in the natural hosts of the disease (17, 33–39). Whether combining H with other PPRV antigens (such as the fusion protein F) will improve the vaccination strategy remains to be fully clarified. Protection assays against challenges with virulent PPRVs were performed in sheep or goats immunized with some of these potential vaccines capable of successfully inducing immune responses: goats have been protected by PPRV-F delivery through capripoxvirus vector (40), and also by PPRV-H or PPRV-F proteins expressed from vaccinia-based vectors (34). A recombinant Newcastle disease virus expressing PPRV-H was also shown to induce protection against PPRV challenge in goats (41), which could potentially be long-lasting (42). Recombinant replication defective adenoviruses expressing H- and/or F-PPRV proteins can protect against virulent PPRV challenge in sheep (17) and goats (35, 43). Elucidating the exact antigenic determinants that provide PPRV protection will surely help design more rational recombinant vaccine approaches. The choice of recombinant vector for PPRV protein expression as an immunogen remains an open question. In the absence of direct comparison studies between vaccine vectors, it is difficult to speculate which are more promising, although ease of production and stability will be critical aspects for potential commercialization.

Overall, our data show the potential of BoHV-4-A-PPRV-H-ΔTK as a DIVA multistrain vaccine and confirm the versatility of recombinant BoHV-4 as a vaccination platform. This is the first time that sheep have been immunized with a BoHV-4-based vector vaccine candidate. BoHV-4-A-PPRV-H-ΔTK is a novel PPRV vaccine candidate that provides protection against virulent PPRV challenge, overcoming immune suppression and may be combined with other constructs such as adenoviruses if needed. The present work highlights the potency of BoHV-4 recombinant viral vector as an antigen delivery system that could eventually help in PPRV eradication programs.

## Data Availability Statement

The raw data supporting the conclusions of this article will be made available by the authors, without undue reservation.

## Ethics Statement

The animal study was reviewed and approved by the Committee on the Ethics of Animal Experiments of the Spanish Instituto Nacional de Investigación y Tecnología Agraría y Alimentaria (INIA) and the National Animal Welfare Committee (PROEX 032/19).

## Author Contributions

JR, NS, GD, and VM designed the experiments and directed the work. DR-M, VF, FM, LR, JR, NS, and VM performed the experiments. GD, JR, and VM wrote the manuscript. All authors contributed to the article and approved the submitted version.

## Funding

This work was funded mainly by a TNA (Translational Access Activities) from VetBioNet INFRAIA-731014 from the EU-H2020. The study also received the following grants and funding: AGL2015-64290R and RTI2018-094616-B-100 from the Ministerio de Ciencia (Spain), grant S2018/BAA-4370-PLATESA2 from Comunidad de Madrid (Fondo Europeo de Desarrollo Regional, FEDER), and internal funding from Parma University.

## Conflict of Interest

The authors declare that the research was conducted in the absence of any commercial or financial relationships that could be construed as a potential conflict of interest.

## Publisher’s Note

All claims expressed in this article are solely those of the authors and do not necessarily represent those of their affiliated organizations, or those of the publisher, the editors and the reviewers. Any product that may be evaluated in this article, or claim that may be made by its manufacturer, is not guaranteed or endorsed by the publisher.

## Supplementary Material

Supplementary Figure 1 MTT assay comparing OFLs growth with medium containing 10% of FBS and the same number of OFLs growth with medium containing 10% of sheep serum, at different time points (24, 48 and 72 hours). In **(A)** absolute values are expressed as optical density (O.D.), whereas in **(B)** the same data were normalized and expressed as percentage of cell growth, where the cell growth with FBS was considered equal to 100% of growth. The data presented are the means ± standard errors of triplicate measurements.

Supplementary Figure 2 Flow chart of the strategy used to produce BoHV-4-A-PPRV-H-ΔTK and BoHV-4-A-ΔTK free of bovine antigens.
