# Supplementary figures and images for: Immunization With Bovine Herpesvirus-4-Based Vector Delivering PPRV-H Protein Protects Sheep From PPRV Challenge
Source: Front Immunol. 2021 Sep 14;12:705539. doi: 10.3389/fimmu.2021.705539 (PMC8476865; doi:10.3389/fimmu.2021.705539)

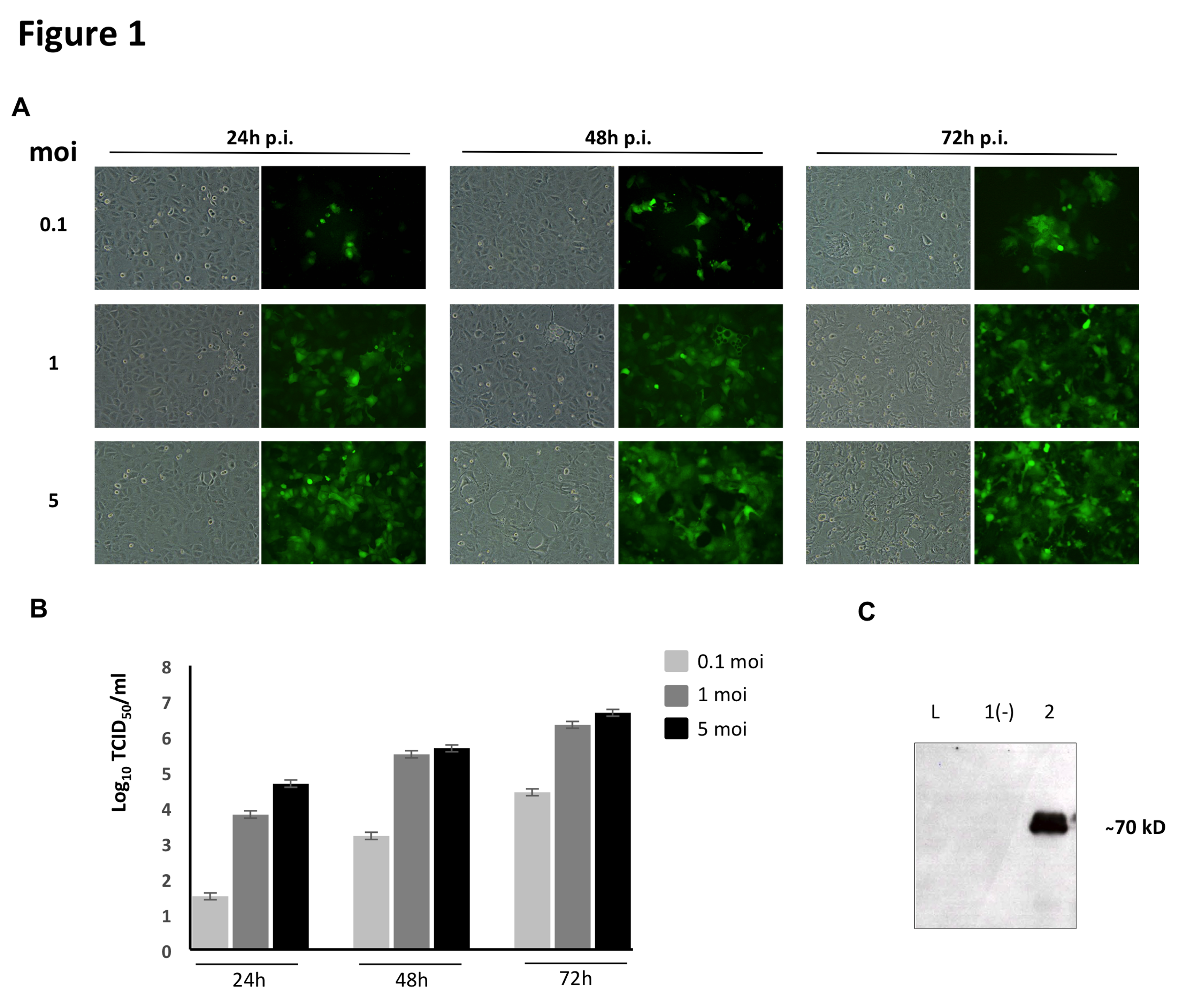

Supplement: Supplementary Figure 1 — MTT assay comparing OFLs growth with medium containing 10% of FBS and the same number of OFLs growth with medium containing 10% of sheep serum, at different time points (24, 48 and 72 hours). In (A) absolute values are expressed as optical density (O.D.), whereas in (B) the same data were normalized and expressed as percentage of cell growth, where the cell growth with FBS was considered equal to 100% of growth. The data presented are the means ± standard errors of triplicate measurements. [file Image_1.tif]
