# Supplementary material for: Immunization With Bovine Herpesvirus-4-Based Vector Delivering PPRV-H Protein Protects Sheep From PPRV Challenge
Source: Front Immunol. 2021 Sep 14;12:705539. doi: 10.3389/fimmu.2021.705539 (PMC8476865; doi:10.3389/fimmu.2021.705539)

Supplementary Figure S2

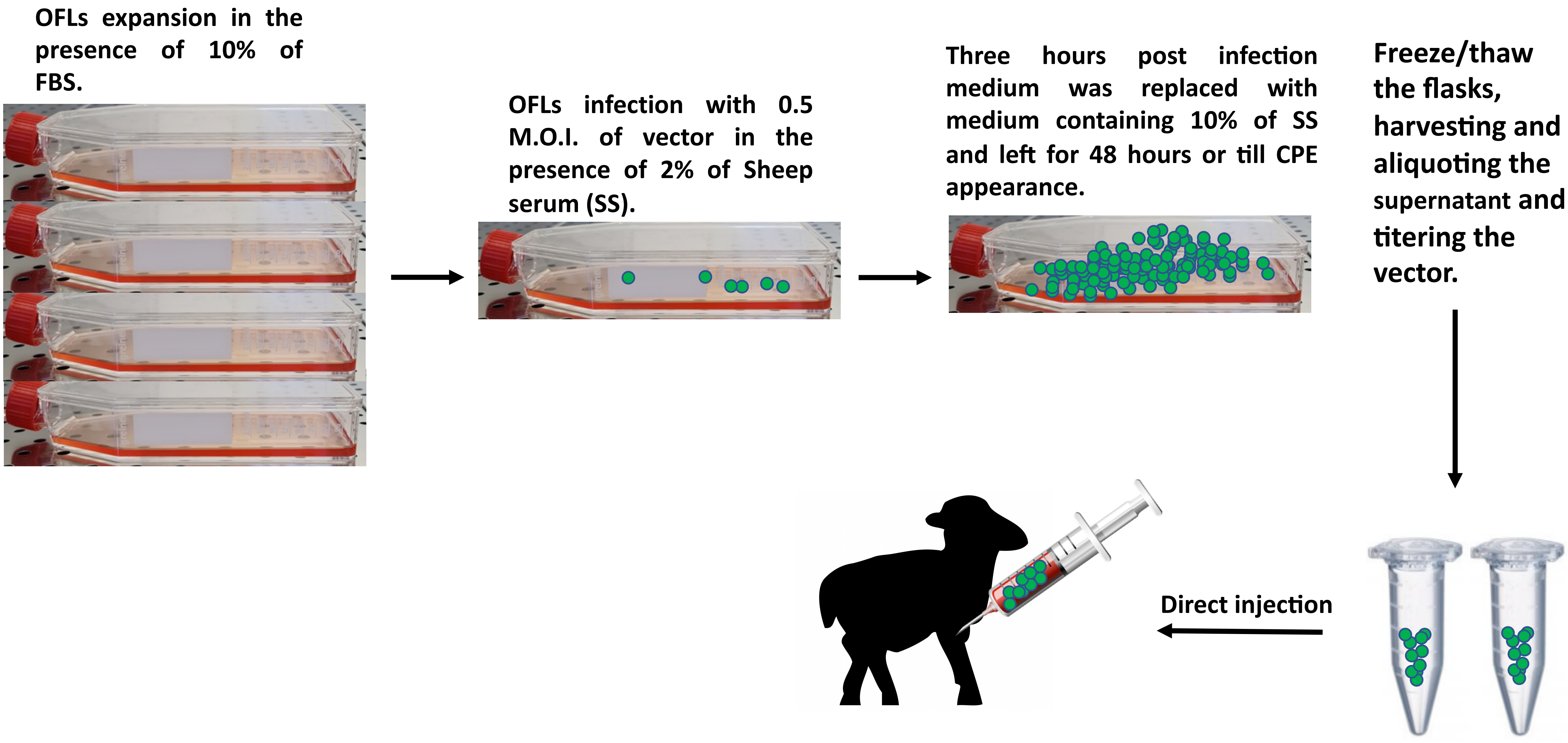

Supplement: Supplementary Figure 2 — Flow chart of the strategy used to produce BoHV-4-A-PPRV-H-ΔTK and BoHV-4-A-ΔTK free of bovine antigens. [file Image_2.pdf]
